# An examination of the mediating role of maladaptive emotion regulation strategies in the complex relationship between interpersonal needs and suicidal behavior

**DOI:** 10.3389/fpsyt.2024.1301695

**Published:** 2024-06-07

**Authors:** Hamed Abdollahpour Ranjbar, Michael Bakhshesh-Boroujeni, Sepideh Farajpour-Niri, Issa Hekmati, Mojtaba Habibi Asgarabad, Mehmet Eskin

**Affiliations:** ^1^ Department of Psychology, College of Social Sciences and Humanities, Koç University, Istanbul, Türkiye; ^2^ Department of Psychology, University of Tabriz, Tabriz, East Azerbaijan, Iran; ^3^ Department of Psychology, Shiraz University, Shiraz, Fars, Iran; ^4^ Department of Psychology, University of Maragheh, Maragheh, Iran; ^5^ Department of Psychology, Norwegian University of Science and Technology, Trondheim, Norway

**Keywords:** suicidal behavior, interpersonal needs, emotion, cognitive emotion regulation strategies, structural equation modeling

## Abstract

**Background:**

Studies have shown that psychological factors, notably interpersonal needs and emotion regulation, play a significant role in suicidal behavior. Interpersonal needs are significant contextual components that affect emotion regulation and contribute to a wide range of dysfunctional behaviors, such as suicidal behavior. It has been postulated that emotion regulation mediates the associations between proximal and distal risk factors of suicidal behavior.

**Method:**

The sample consisted of 340 community-dwelling individuals (62.5% women; SD = 0.48) with an age range of 18 through 55 (M = 30.23; SD = 8.54) who completed the interpersonal needs questionnaire, the suicide behaviors questionnaire-revised, and the cognitive emotion regulation questionnaire. The Structural Equation Modeling (SEM) approach was utilized to evaluate a mediation model.

**Results:**

The findings indicate that interpersonal needs (i.e., perceived burdensomeness r = .55, p <.01 and thwarted belongingness r = .25, p <.01) and putatively maladaptive cognitive emotion regulation strategies (i.e., self-blame; r = .38, p <.01, catastrophizing; r = .55, p <.01, rumination; r = .40, p <.01, and other blame; r = .44, p <.01) have strong associations with suicidal behavior, and these strategies have a mediating effect on the association between interpersonal needs and suicidal behavior.

**Conclusions:**

Our findings show that contextual-interpersonal needs, which underpin suicidal behavior, are significantly influenced by maladaptive emotional processes. Thus, therapeutic outcomes might be enhanced by focusing on the content of the associated cognitions and trying to reduce maladaptive regulatory processes like rumination and catastrophization.

## Introduction

Suicide is a highly complicated and multidimensional problem (1) that has significant public health implications globally for both clinical and nonclinical groups (2, 3). According to estimates from the World Health Organization (WHO), suicide is one of the leading causes of death worldwide, taking the lives of over 700,000 people annually (4).

Prospective suicide research has demonstrated that psychological factors are the most important indicators of suicidal behavior [e.g., 5–8], with interpersonal needs (9, 10) and emotion regulation (ER) (11–13) among the most prominent ones. Interpersonal needs are one of the critical contextual elements that affect human behavior and constitute a significant portion of dysfunction throughout mental illnesses [e.g., 10, 14]. Joiner’s (2005) Interpersonal Theory of Suicide is a prominent conceptual paradigm in this regard (15). As per Joiner’s theory, suicide desire is featured by two distinct sets of interpersonal cognitions: thwarted belongingness and perceived burdensomeness. Thwarted belongingness signifies an individual’s belief that they lack meaningful relationships with others, whether if nobody appears to care or even because others care but cannot empathize with the individual’s actual status and complex feelings (e.g., an individual who experienced sexual harassment and rape). Perceived burdensomeness refers to an individual’s belief that they make no substantial contributions to the world but rather serve as an encumbrance to others. Empirical evidence on these factors has shown their prognostic value for suicidal behavior, with many studies revealing that the two-way interaction of perceived burdensomeness and thwarted belongingness explains a significant portion of suicidal desire (10, 16, 17).

The ER capacity is crucial for psychological development and functioning and is defined as strategies used to affect the experience and modify emotions, which might also entail mechanisms such as suppression and cognitive reappraisal of a distressing incidence (18). Linehan (1993) described “emotion dysregulation as excessive emotional vulnerability, heightened reactivity to emotion cues, and slow return to emotional baseline” (19). Notably, several models of psychopathology have shown strong links between psychopathology and emotion dysregulation (20–22). In fact, emotion dysregulation has been linked to a number of psychiatric conditions, namely depression (23, 24), non-suicidal self-injury [NSSI; 25], and, most importantly, suicidal behavior (11–13). Considering the transdiagnostic (26) and context-dependent [e.g., 27] nature of emotion dysregulation, extant theories explaining mechanisms of its escalation in severe problem behaviors (such as suicidal ideation and attempt) are critical for inspection.

Recent contemplations in the ER literature point to the necessity of considering the context (28–31), which has been largely overlooked in studies of ER and its associations with psychopathologies (28). All emotions serve an interpersonal function (32, 33); thus, emotion dysregulation may be a critical feature of the interpersonal theory of suicide paradigm. Also, it has been linked as a potential risk factor for the theory’s key elements, namely, suicide desire. Existing research indicates an association between greater levels of suicide desire and increased levels of emotion dysregulation (11–13). Individuals who are easily dominated by emotion dysregulation (i.e., poor ER capacities) can be more vulnerable to suicidal desire (16). Consequently, emotion dysregulation may be a significant risk factor for the urge to terminate one’s life.

In the literature, cognitive strategies, including rumination, self-blame, other-blame, and catastrophizing, consistently have been associated with negative emotions like depression, anxiety, stress, and anger [34, p 1046]. Accordingly, we will consider them maladaptive ER strategies in this study. Though evidence is sparse, these strategies may be the mechanisms by which hindered interpersonal needs increase the likelihood of suicidal behavior. For each putatively maladaptive ER strategy, the phenomenology is described below, as well as their probable associations to suicidal thought and behavior and how each strategy may pertain theoretically to perceived burdensomeness and thwarted belongingness.

The propensity to react to distress by dwelling/concentrating upon the origins and repercussions of one’s difficulties without engaging in active problem-solving is known as rumination [See The Response Style Theory; 32]. Depressive rumination is a contemporaneous/prospective indicator of depression [e.g., 35, 36] and suicidal ideation and behavior [e.g., 37, 38]. Recent research has consistently highlighted the association between rumination and suicidal ideation and/or behavior, regardless of methodology, samples, or measurements (39). Depressive rumination has been implicated in deteriorating well-being and feelings of life satisfaction among older adults by escalating perceived burdensomeness (40). Moreover, significant interactions have been observed between brooding (a subtype of rumination) and thwarted belongingness, as well as between perceived burdensomeness, brooding, and gender on suicide risk (41).

Catastrophizing refers to particular thoughts accentuating the dread of what you have experienced (34). Previous research has indicated positive associations between catastrophizing and suicidal behavior [e.g., 12, 42], although this concept’s association with suicidal behavior has been studied principally in the context of pain catastrophization. Consequently, there is a well-established link between suicidality and pain catastrophizing. Only Shim et al. explored the association between perceived burdensomeness and catastrophizing and suicide concerning interpersonal needs (43). They demonstrated that the link between pain catastrophizing and suicide was mediated by perceived burdensomeness. However, this cognitive distortion (i.e., catastrophizing) seems to play a more critical and neglected role in perceiving obstructed interpersonal needs. As defined in the literature, cognitive distortion is an excessive or unreasonable thinking pattern that contributes to the genesis or maintenance of psychopathologies (44). It is found that cognitive distortions (e.g., negative evaluation of self) are associated with suicidal thinking (45). Catastrophizing, as an exaggerated, maladaptive ER strategy, can be evident in the perception of burdensomeness and thwarted belongingness as significant cognitive distortions of interpersonal needs. These distortions may be perceived unduly, contributing to the amplification of suicidal thoughts and behavior. As a result, the possible associations between interpersonal needs and catastrophizing may be both detrimental and conducive to suicidal ideation/behavior.

Self-blame is defined as the act or thoughts of blaming yourself for what you have gone through (34). According to empirical evidence, prolonged negative thinking, such as self-blame, might increase the likelihood of suicidal thoughts and behaviors (46, 47) and also other psychopathologies [e.g., depression; 47]. Findings from MMPI profiles of individuals lost to suicide suggest that excessive self-blaming is a self-defeating behavior that increases the possibility of completed suicide compared to individuals who died by other causes (48). This could be elucidated by drawing on Baumeister’s contention that suicide might be seen as an attempt to flee from painful self-awareness. When individuals encounter situations that significantly deviate from their personal expectations or societal standards, it can trigger a cascade of reactions, potentially leading to avoidant behaviors and, at the extreme, suicidal behavior. Recognizing their shortcomings leads to negative emotional responses, prompting a desire to avoid both self-awareness and the emotional distress it entails (49). Also, it has been demonstrated that self-blame might lead to chronic distress and suicidal behavior among sexual assault survivors (50). The interpersonal theory of suicide proposes that self-blame and a sense of being neglected by others are elements that contribute to thwarted belongingness and perceived burdensomeness [for more details, see; 7, 50]. Indeed, perceived burdensomeness has been conceptualized as comprising a degree of self-hatred, as evidenced by the existence of self-blame and low self-esteem.

Other blame refers to the idea of blaming others or your surroundings for the experience you have had (34). Other blame has received little research attention among individuals at high risk of suicide thus far. Horesh et al., in their investigation of suicide risk and coping styles, found that psychiatric patients utilized suppression and other blame coping styles more frequently than other maladaptive strategies (51). The majority of evidence of blaming others as a maladaptive ER strategy contributing to suicidal behavior has been revealed in the retrospective suicide notes. Studies analyzing suicide notes found that the most prevalent reasons for blaming others were being wrongfully accused, being stubborn, and feelings of disagreement/hatred, respectively (52), and three major themes in suicide notes were found, indicating a failed relationship and an attempt to escape from this situation (53). These are associated with interpersonal needs, comprising impeded belongingness, alienation, and burdensomeness, all of which contribute to blaming oneself/others for failures and possibly lead to suicidal thoughts.

### Bonding interpersonal needs and emotion regulation and current study

According to earlier meta-analytic findings, which indicated larger effect sizes for maladaptive ER strategies (e.g., rumination) in psychopathology and abnormal behavior and smaller effect sizes for adaptive ER strategies (e.g., reappraisal) (20), we solely took into account maladaptive ER strategies in the current investigation and our model. We attempted to associate and position variables of interest according to the process model of emotion [emotion regulation as an information processing model; 54] and the heuristic transdiagnostic model of ER (55). The authors delineated this heuristic by concentrating on the supposedly maladaptive ER strategy of rumination. They hypothesized, particularly, that in the setting of a perceived threat, rumination possibly contributes to the onset of anxiety disorders, but when rumination meditates in a high sensitivity to alcohol context, it can contribute to substance abuse or, when mediating sadness-loss-derived mood can lead to depression. Using this paradigm as a foundation, we also considered that maladaptive ER strategies in the context of hindered interpersonal needs (i.e., perceived burdensomeness and thwarted belongingness) would possibly lead (i.e., mediate) to suicidal behavior. It is important to mention that we considered interpersonal needs as contextual factors of suicide and maladaptive ER strategies as the processes through which these cognitive susceptibilities/distortions are mediated toward suicidal behavior.

We hypothesized that the proposed model for associations of interpersonal needs with suicidal behavior through the mediating role of maladaptive ER strategies would fit the data well. Specifically, we hypothesized that i) perceived burdensomeness would be positively associated with suicidal behavior, ii) perceived burdensomeness indirectly (through maladaptive ER strategies) would be associated with the suicidal behavior, iii) thwarted belongingness would be positively associated with suicidal behavior, and iv) thwarted belongingness indirectly (through maladaptive ER strategies) would be associated with suicidal behavior.

In conclusion, the interpersonal theory of suicide emphasizes the role that perceived burdensomeness and thwarted belongingness have triggering roles in suicide behaviors, and emotion dysregulation may play a mediation role in these relationships. Rumination, catastrophizing, self-blame, and other blame are instances of maladaptive ER strategies that may act as conduits via which unmet interpersonal needs influence suicidal behavior. Through maladaptive ER strategies, this study attempts to evaluate these correlations and hypothesizes the direct and indirect impacts of thwarted belongingness and perceived burdensomeness on suicidal behavior. By elucidating these relationships, we seek to enhance understanding of suicide risk factors and inform more effective prevention and intervention strategies. This sets the stage for our methodology section, where we detail our approach to examining these complex dynamics empirically.

## Method

### Participants

The current study is a cross-sectional study using a sample of 345 individuals (62.5% women; SD = .48) with an age range of 18 through 55 (M= 30.23; SD = 8.54) from Iran’s capital, Tehran. During the data collection phase, 18 (5%) participants were excluded from the research due to prior diagnoses (self-reported) of bipolar disorder, serious head injury, depressive disorder, personality disorders, anxiety disorders, or other psychiatric conditions. A total of 340 individuals completed the online surveys. After screening for missing values, five (1.4%) incomplete/corrupted data sets were eliminated from the data pool. Finally, the analysis included 128 men’s (37.5%) and 212 women’s (62.5%) data. The age range was 18–55 for men (M = 24.69) and women (M = 24.41). In this sample, education was classified into six levels: high school or lower (n = 4, 1.1%), diploma (n = 67, 18.9%), associate (n = 22, 6.2%), bachelor’s (n = 153, 43.1%), master’s (n=86, 24.2%), and Ph.D. (n = 23, 6.5%).

### Measures

#### The Suicide Behaviors Questionnaire-Revised

**Figure 1:**
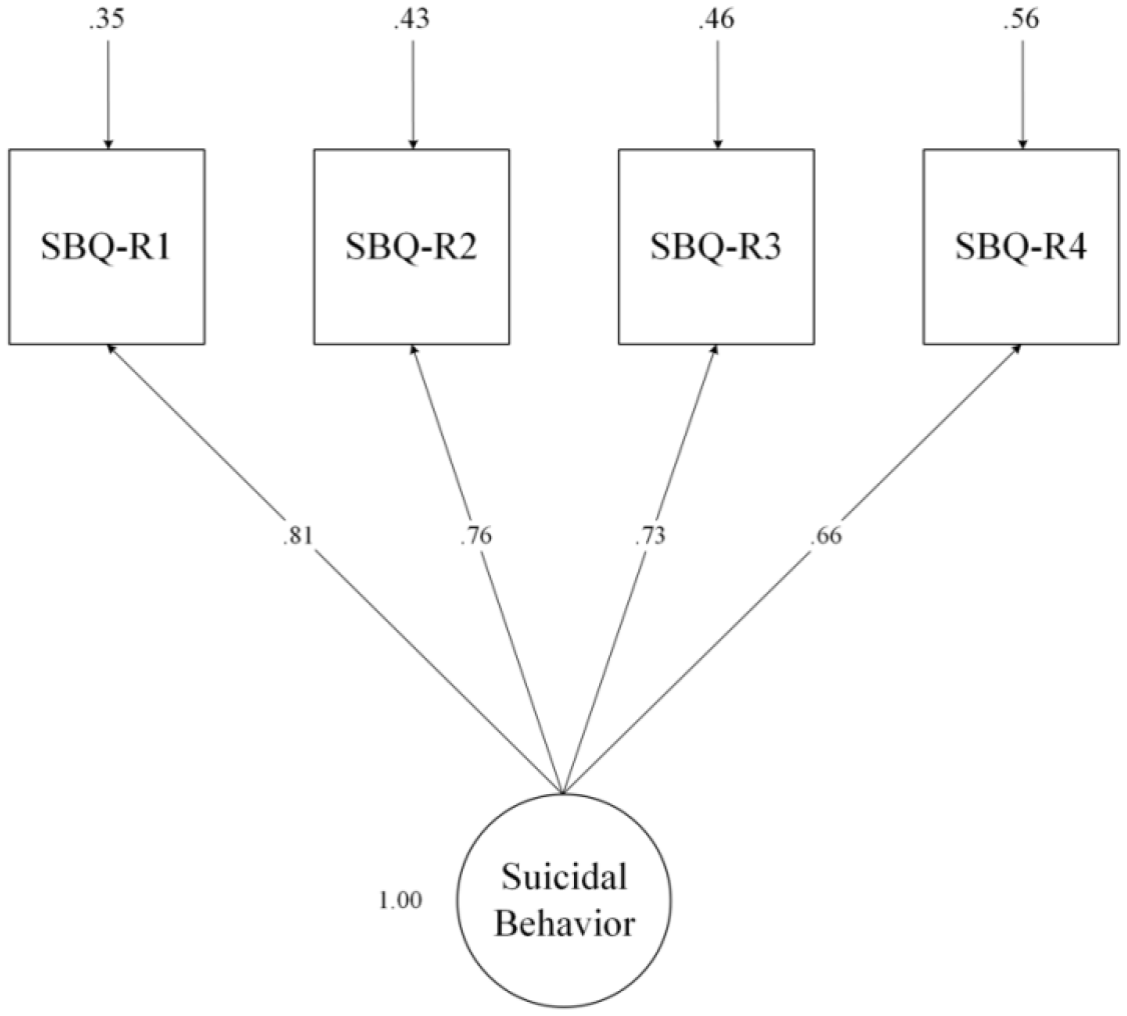
One-factor first-order confirmatory factor analyses of Suicide Behaviors Questionnaire-Revised (SBQ-R).

[SBQ-R; 56] is a reliable questionnaire for identifying individuals at risk of suicide-related thoughts and behavior. The acceptable and appropriate sensitivity (80%) and specificity (91%) of the measure among psychiatric inpatients have been reported (56). Also, it has been shown that its validity and reliability are robust among different cultures and languages, and it is a valid questionnaire for assessing suicide-related thoughts and behavior (57). The questionnaire is comprised of four items, each assessing a distinct aspect of suicidal behavior and thought. Item 1(*have you ever thought about or attempted to kill yourself)?* assesses lifetime suicidal ideation or suicide attempts, item 2 (*how often have you thought about killing yourself in the past year*)*?* assesses the frequency of suicidal ideation over the last year, item 3 (*have you ever told someone that you were going to commit suicide, or that you might do it)?* assesses the risk of suicide attempt, and eventually, item 4 (*how likely is that you will attempt suicide someday*)? measures the self-reported probability of suicidal behavior in the future. The overall score ranges from 3 to 18. Suicidality is regarded as high when the score is greater than 7 in the general population and 8 in individuals with mental illnesses (56). For the Persian version of the SBQ-R, Amini-Tehrani et al. reported composite reliability and average variance extracted values of .87 and .63, respectively (58). Cronbach’s alpha for SBQ-R was .78 in the current study. Confirmatory factor analyses in this study showed that the one-factor first-order model fit the data satisfactorily [χ^2^(2)= 2.08, p = .35; CFI= 1; TLI= 1; RMSEA = .011, 90% CI (.001 to.106); SRMR= .014, **Figure 1**].

#### The Interpersonal Needs Questionnaire

**Figure 2:**
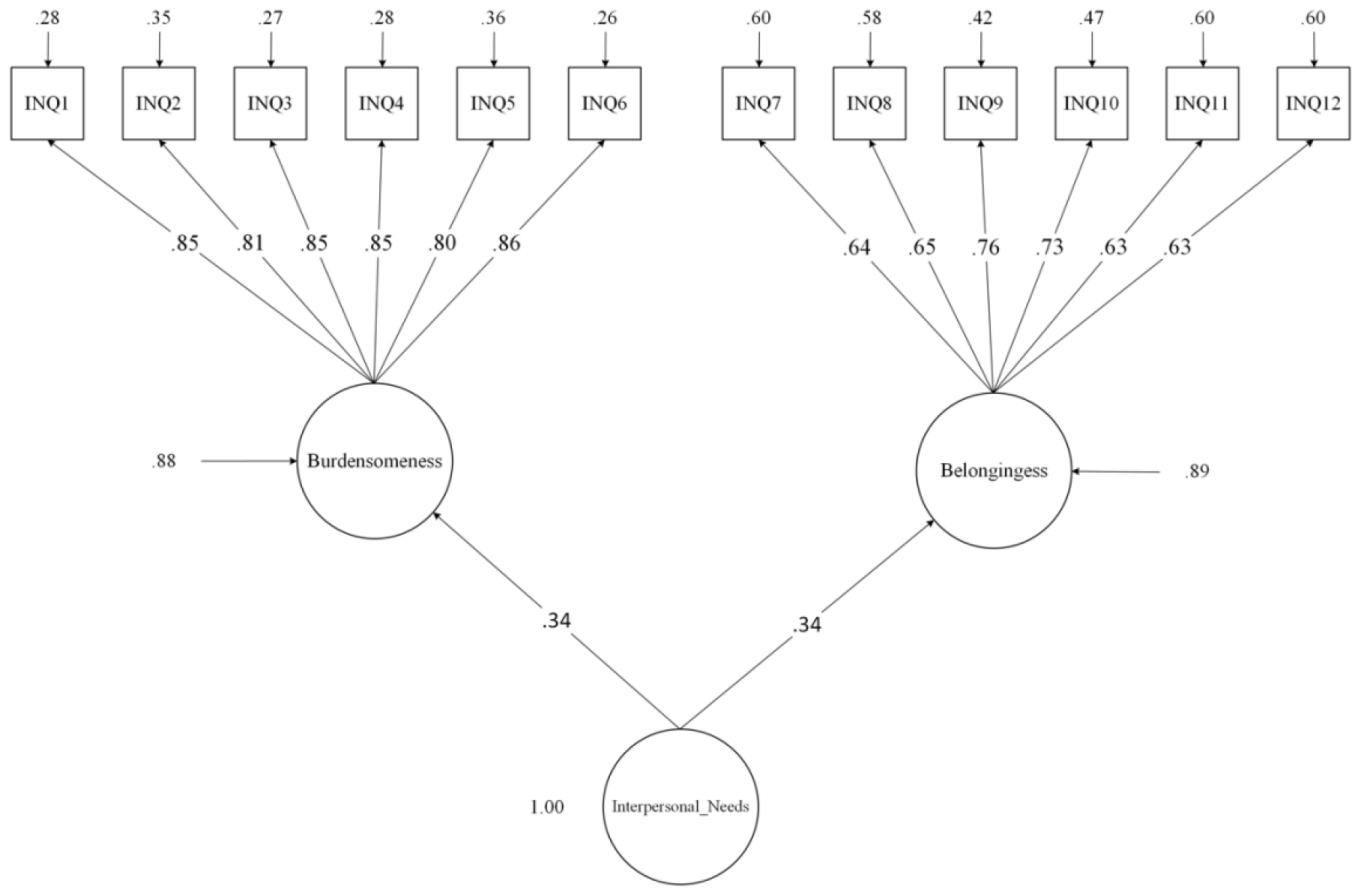
Two-factor first-order and one-factor second-order confirmatory factor analyses of the Interpersonal Needs Questionnaire (INQ).

[INQ; 17, 59] is developed for application by researchers looking into the etiology of suicidal ideation and behavior and clinicians looking for a suicide risk estimation model based on the interpersonal theory of suicide. It is used in both research and therapeutic settings to assess the themes of thwarted belongingness (e.g., “*These days other people care about me*”) and perceived burdensomeness (e.g., “*These days I feel like a burden on the people in my life*”). The original INQ comprises 25 items, although other abbreviated versions (i.e., 10, 12, 15, 18) have been developed thus far. According to Hill and Pettit, the 15 and 10-item versions have been reported to have the highest internal consistency and congruence with exploratory factor analysis models (60). To answer the INQ, respondents indicate the extent to which each question is accurate to them lately (on a 7-point Likert scale). Higher ratings indicate greater thwarted belongingness and perceived burdensomeness. Good reliability (α = .90) has been reported for this measure (54, 59). In the Persian version of INQ, three items (9, 11, and 12) were excluded from the questionnaire due to low factor loading values. Two-factor structure and good reliability results (Cronbach’s α >.60) have been reported for both factors in the Persian version (61). In this study, Cronbach’s α for total INQ was.82, For perceived burdensomeness subscale.93, and thwarted belongingness subscale was.83. Confirmatory factor analyses in the current study showed that the two-factor first-order and one-factor second-order hierarchical model fit the data satisfactorily [χ^2^(52)= 83.35, p = .004; CFI= .98; TLI= .97; RMSEA = .04, 90% CI (.024 to.057); SRMR= .035, **Figure 2**].

#### The Cognitive Emotion Regulation Questionnaire

**Figure 3:**
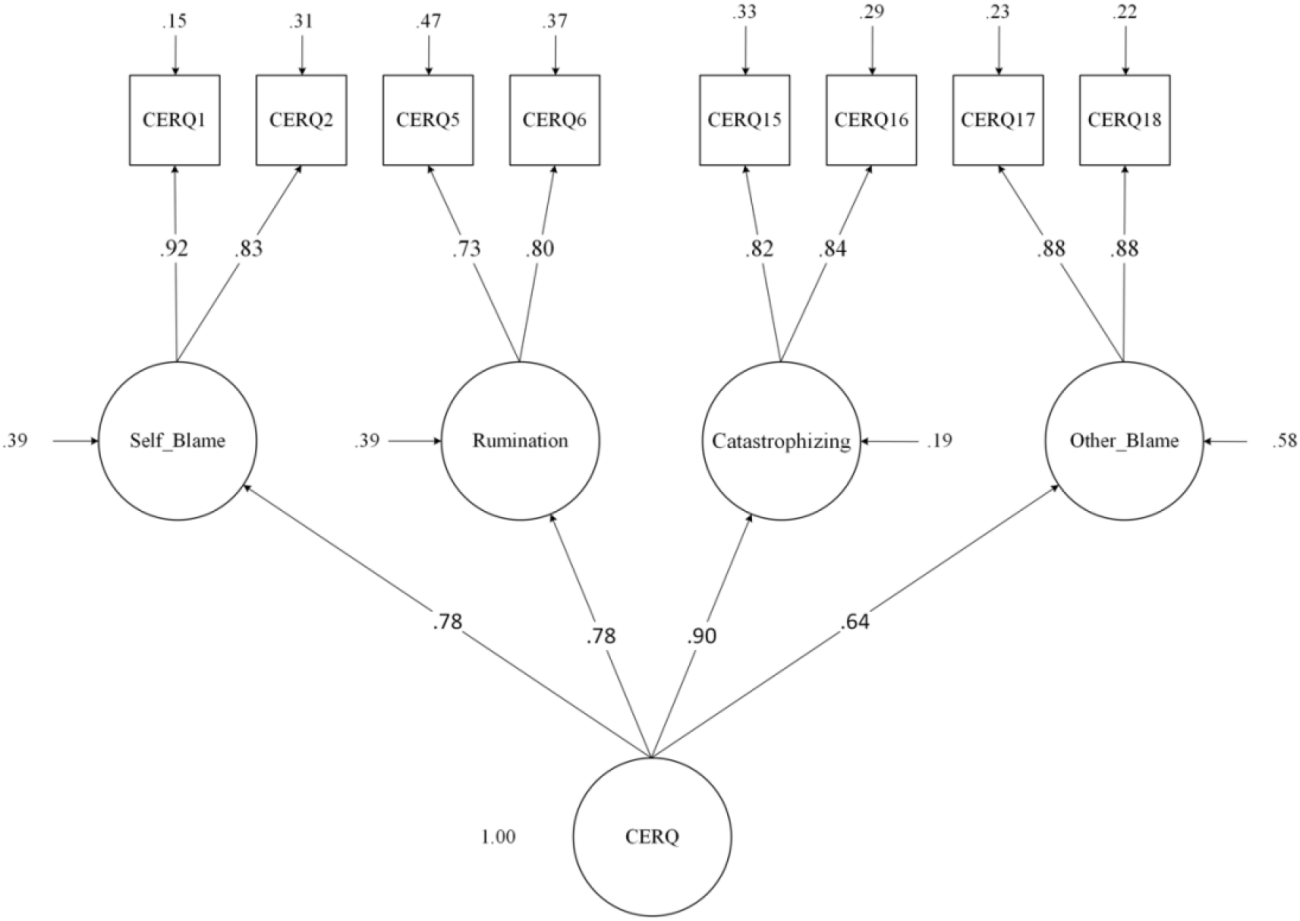
Four-factor first-order and one-factor second-order confirmatory factor analyses of the Cognitive Emotion Regulation Questionnaire (CERQ-18)(maladaptive strategies).

[CERQ-18; 34] was established to evaluate cognitive emotion regulation strategies following stressful life situations (62). It is comprised of nine dimensions, each including four items: *positive reappraisal, acceptance, positive refocusing, perspective taking, perspective taking, blaming others, catastrophizing, rumination, and self-blame.* It includes a 5-point Likert scale ranging from 1 “(almost) never” to 5 “(almost) often,” with scores between 4 to 20, with higher scores betokening a higher usage of that particular strategy. We used the short Persian form of the CERQ, which has 18 items (2 items for each dimension) and has been demonstrated to have good psychometric properties (Cronbach’s spanned between.64 to.92), equivalent to the long version’s (63). As the literature suggests that maladaptive strategies might mediate the effects on psychopathology and behavioral disorders [for review see; 22], we employed the maladaptive ER strategies subscales in our investigation. Overall Cronbach’s alpha for the CERQ was .75, with the following for each subscale: Self-blame .78, Rumination .69, Catastrophizing .76, and Other-blame .86. Confirmatory factor analyses in this study showed that the four-factor first-order and one-factor second-order hierarchical model fit the data satisfactorily [χ^2^(17) = 94.16, p <.001; CFI= .9; TLI= .83; RMSEA = .11, 90% CI (.091 to.14); SRMR= .022, **Figure 3**].

### Procedure

Data was collected via Google Docs and a social media advertising link, and the data-collecting period spanned from September to December 2022. To ensure the integrity of our data, we implemented a series of rigorous measures. These included monitoring IP addresses and utilizing CAPTCHA verification to deter automated responses, as well as restricting submissions to one per device through Google Forms. To further enhance data quality, we employed data-cleaning algorithms to identify and remove duplicates. Additionally, randomized response techniques were integrated into our survey design to minimize response bias and encourage genuine responses. We provided explicit instructions to participants, emphasizing the importance of truthful reporting. Furthermore, we offered support services and contact information for individuals in need of assistance, including a debriefing statement to provide guidance post-survey completion. These comprehensive efforts aimed to mitigate risks to data quality and ensure the authenticity and reliability of our findings. Participants were informed on the consent form that their responses would be kept confidential and processed anonymously and that their participation was entirely voluntary, with the opportunity to discontinue at any point. The project complied with the Helsinki Declaration, was approved by the University of Maragheh, and was subject to the local council for ethics in clinical research evaluation.

### Statistical analysis procedure

**Figure 4:**
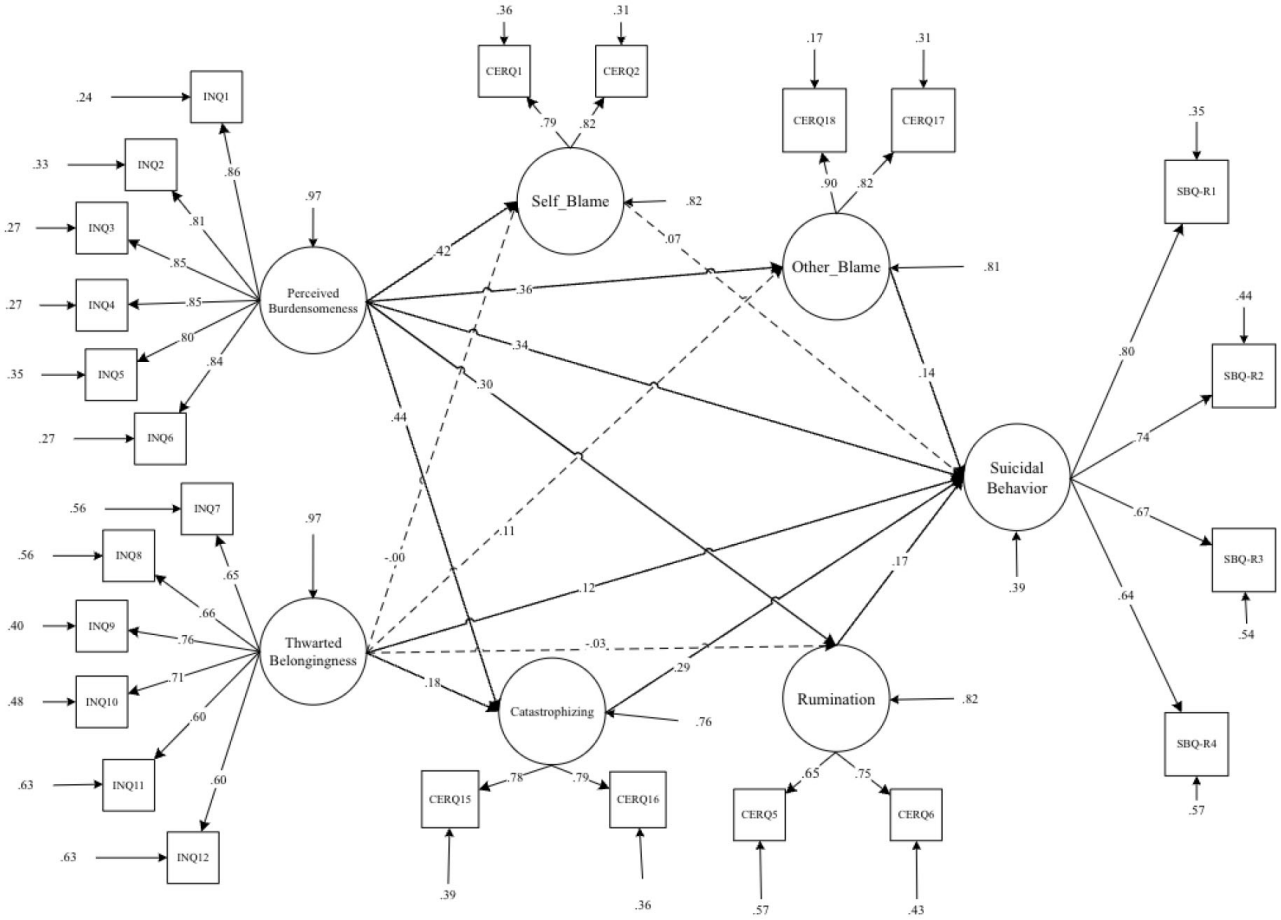
Path diagram of the hypothesized structural model for interpersonal needs, maladaptive cognitive emotion regulation strategies, and suicidal behavior.

G*Power software (64) was employed to determine the sample size needed to attain sufficient power (.80) for a small effect size (.02) for the conceptual model based on the number of predictor variables (**Figure 4**). Statistical software SPSS 28.0.1 (65) and Mplus 8.8 (66) were used to analyze the data, and the following five procedures were then followed:

**1^st^ Step.** For the preliminary analysis, all variables were inspected for missing values, outliers, and assumptions (67). The assumption of normality was marginally supported by the questionnaire subscales, which exhibited no discernible skewness. The sample size was appropriate; thus, no changes were necessary (68).

**2^nd^ Step.** Using Cronbach’s alpha, the internal consistency of the SBQ-R, INQ, and CERQ-18 was assessed (69, 70). Here, a correlation value of at least .70 was considered to be a sufficient degree of item internal consistency (71). To corroborate the theoretical construct’s breakdown into a certain number of subcomponents, second-order confirmatory factor analysis was performed on each measure used in this study.

**3^rd^ Step.** To test *a priori* models of the scales’ factorial validity, we used CFA employing Weighted Least Squares Mean-Variance (WLSMV) (72, 73). The “goodness-of-fit” of the models was then assessed using the statistical tests and index values listed below: the Comparative Fit Index (CFI) where coefficients > .95 indicating good fit (74), Chi-square/degree of freedom CMIN/DF—where values < 5.0 indicate good fit (75), the Tucker-Lewis index (TLI), coefficients > .95 (76) and the Root Mean Square Error of Approximation (RMSEA ≤ .06 suggests good fit) also indicated good fit (75, 77). Additionally, the fit indices of all models and the multivariate skewness in our data were adjusted using the Satorra-Bentler scaled chi-square test statistic, which corrects for non-normality and improves model fit assessment accuracy (78).

**Figure 5:**
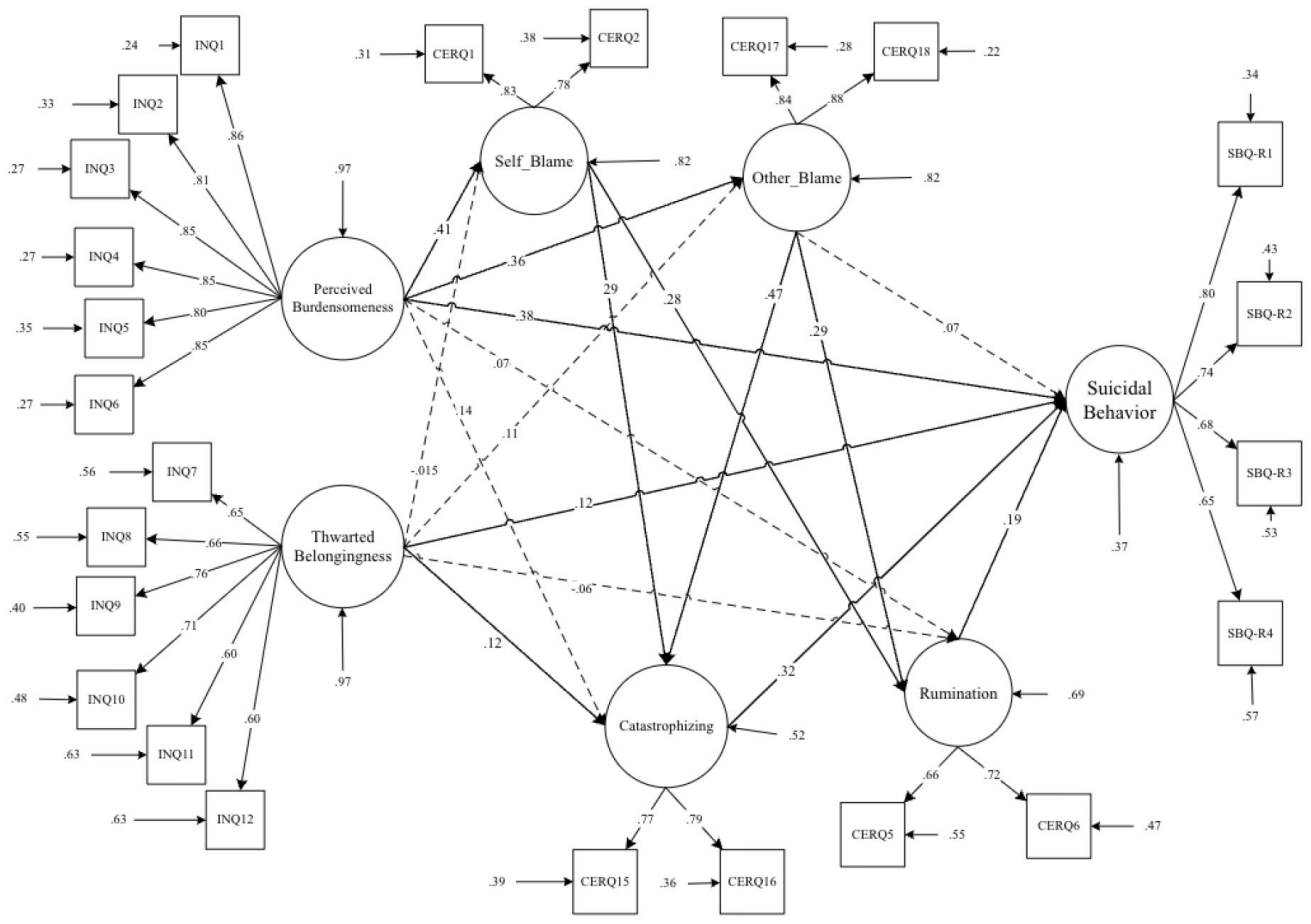
Path diagram of the Modified structural model for interpersonal needs, maladaptive cognitive emotion regulation strategies, and suicidal behavior.

**4^th^ Step.** In the conceptual model (**Figure 4**), the interpersonal needs indicators (perceived burdensomeness and thwarted belongingness) were included as exogenous variables, while suicidal behavior indicators were treated as endogenous variables. Additionally, maladaptive emotion regulation (ER) strategies such as self-blame, rumination, catastrophizing, and other blame were included as mediators. Originally, the model included direct pathways from ‘self-blame’ and ‘other-blame’ to ‘suicidal behavior.’ However, in the modified model, ‘self-blame’ and ‘other-blame’ were regressed on ‘rumination’ and ‘catastrophizing thinking.’ This adjustment was made to account for the indirect effects of rumination and catastrophizing thinking on suicidal behavior through the mediation of blaming self/other attributions. By regressing self-blame and other blame on rumination and catastrophizing, the model suggests that these cognitive processes may contribute to higher levels of self-blame and other blame. Consequently, this increase in blame attributions could elevate the risk of experiencing suicidal behavior. Therefore, addressing rumination and catastrophizing may be crucial, as they indirectly influence suicidal behavior through their impact on blame attributions (**Figure 5**).

**5^th^ Step.** Traditional indirect, direct, and total effects—all effects employed in conventional mediation research—as well as their standard errors, are obtained using the MODEL INDIRECT command in Mplus 8.8 (79). It should be noted that using maximum likelihood estimation (MLR) with robust standard errors (73), the mediator model was evaluated in models 1 and 2.

## Results

The descriptive statistics and bivariate correlations between interpersonal needs, suicide behaviors, and cognitive emotion regulation indicators are presented in **Table 1**. As evident in **Table 1**, all bivariate correlations related to the observed indicators are significant. Accordingly, it indicates that the conceptual model has solid conceptual and statistical underpinnings for investigating the hypothesized causal model for mediation analysis. As a result, we investigated a latent variable mediation model based on observed variables (**Figures 4**, **5** and **Table 2**).

**Table 1:** Means, standard deviations, and bivariate correlations between interpersonal need, maladaptive cognitive emotion regulation strategies, and suicidal behavior.

_Variable_	_M_	_SD_	_1_	_2_	_3_	_4_	_5_	_6_	_7_	_8_
_1. Suicidal Behavior_	_5.46_	_2.91_	_1_							
_2. Interpersonal Needs Total_	_31.96_	_10.84_	_.52**_	_1_						
_3. Perceived Burdensomeness_	_10.26_	_6.53_	_.55**_	_.68**_	_1_					
_4. Thwarted Belongingness_	_21.69_	_7.99_	_.25**_	_.80**_	_.11*_	_1_				
_5. Maladaptive Cognitive Emotion Regulation Strategies total_	_18.95_	_4.87_	_.62**_	_.38**_	_.46**_	_.13*_	_1_			
_6. Self-Blame_	_4.24_	_1.80_	_.38**_	_.22**_	_.36**_	_.01_	_.66**_	_1_		
_7. Other Blame_	_3.81_	_1.44_	_.44**_	_.31**_	_.34**_	_.14**_	_.66**_	_.21**_	_1_	
_8. Rumination_	_6.32_	_1.80_	_.40**_	_.19**_	_.26**_	_.05_	_.74**_	_.27**_	_.31**_	_1_
_9. Catastrophizing_	_4.57_	_1.76_	_.55**_	_.36**_	_.37**_	_.18**_	_.79**_	_.33**_	_.46**_	_.48**_

∗p <.05, ∗∗p <.01.

**Table 2:** Modification indices for the mediated model of interpersonal needs, maladaptive cognitive emotion regulation, and suicidal behavior.

Model	χ^2^	df	χ^2^/df	CFI	TLI	RMSEA	SRMR	Base model	ΔS-Bχ^2^(Δdf)
M_1_	583.80	304	1.9	.93	.92	.051(.045 -.057)	.082	-	-
M_2_	486.25	301	1.62	.953	.946	.042(.035 -.048)	.067	M1	81.89**(3)

M_1_ = structural equation modeling of pathways from interpersonal needs to suicidal behavior: mediating maladaptive cognitive emotion regulation; M_2_ = in the modified model, ‘self-blame’ and ‘other blame’ were regressed on ‘rumination’ and ‘catastrophizing.’ This adjustment suggests that the levels of self-blame and other blame are influenced by the extent of rumination and catastrophizing experienced by individuals. This approach allowed us to investigate the indirect effects of blame attributes on suicidal behavior through these cognitive processes. χ^2^ = Chi-square, df = degrees of freedom = χ^2^/df = normal chi-square, TLI = Tucker–Lewis index, CFI = comparative fit index, SRMR = standardized root mean square residual, RMSEA = root mean square error of approximation, Δχ^2^ = difference between minus twice log likelihoods between the full and the nested models, ^∗∗^p <.01. In our analysis, age, sex and marital status were included as covariates in both models to account for their potential influence on the outcomes.

### Mediation analyses

The goodness-of-fit results for two nested-mediator models are summarized in **Table 2**. Initially, a theory-driven specified model (M_1_: **Table 2** and **Figure 4**) did not meet the previously specified fitting criteria. Consequently, M_1_ was modified into M_2_. By incorporating direct paths from self-blame and other-blaming to catastrophizing and rumination, the aim was to better capture the relationships between these variables. This adjustment acknowledged the potential for self-blame and other blame to directly influence catastrophizing and rumination, which in turn may impact suicidal behavior (M_2_: **Table 2** and **Figure 5**).

As indicated in **Table 2**, the modified model (M_2_) significantly improved the model fit compared to the original specification (M_1_). The two competitive nested models were evaluated according to the parsimony principle, with M_2_ being identified as the most effective model. This suggests that the incorporation of direct paths from self-blame and other blame to catastrophizing and rumination enhanced the model’s explanatory power and provided a better fit to the data (M_2_: **Table 2** and **Figure 5**).

Results of **Table 3**, **Figure 5** show a significant direct association between perceived burdensomeness and suicidal behavior (β = .38), thwarted belongingness and suicidal behavior (β = .12), rumination and suicidal behavior (β = .19), and finally between catastrophizing and suicidal behavior (β = .32). Findings from **Table 4** suggest that self-blame, other blame, rumination, and catastrophizing have a mediating role in the association between perceived burdensomeness and suicidal behavior (p < .05).

**Table 3:** Standardized direct effects of interpersonal needs and cognitive emotion regulation on suicide behaviors.

Paths	Direct effect	*p*
Perceived Burdensomeness → Suicidal Behavior	**.38**	.001
Thwarted Belongingness → Suicidal Behavior	**.12**	.020
Rumination → Suicidal Behavior	**.19**	.006
Catastrophizing→ Suicidal Behavior	**.32**	.001
Self-Blame → Suicide Behavior	Fixed to zero	–
Other Blame → Suicidal Behavior	.07	.35
Perceived Burdensomeness → Self-Blame	**.41**	.001
Thwarted Belongingness → Self-Blame	-.015	.81
Perceived Burdensomeness → Rumination	.07	.37
Thwarted Belongingness → Rumination	-.06	.41
Self-Blame → Rumination	**.28**	.001
Other Blame → Rumination	**.29**	.001
Perceived Burdensomeness → Catastrophizing	.14	.073
Thwarted Belongingness → Catastrophizing	**.12**	.047
Self-Blame → Catastrophizing	**.29**	.001
Other Blame → Catastrophizing	**.36**	.001
Perceived Burdensomeness → Other Blame	**.36**	.001
Thwarted Belongingness → Other Blame	.11	.094

CI, confidence intervals. Age, sex, and marital status were incorporated as covariates. Significant paths are highlighted in bold. The coefficients are presented in the standardized format.

**Table 4:** Indirect standardized effects of interpersonal needs and maladaptive cognitive emotion regulation strategies on suicidal behavior.

	coefficient	P
Effects from Perceived Burdensomeness to Suicidal Behavior		
**Total**	**.59**	.001
**Total Indirect**	**.22**	.001
**Perceived Burdensomeness → Rumination → Suicidal Behavior**	.014	.37
**Perceived Burdensomeness → Catastrophizing → Suicidal Behavior**	.044	.07
**Perceived Burdensomeness → Other Blame → Suicidal Behavior**	.024	.34
**Perceived Burdensomeness → Self-blame → Rumination → Suicidal Behavior**	.022	.049
**Perceived Burdensomeness → Other Blame → Rumination → Suicidal Behavior**	.019	.043
**Perceived Burdensomeness → Self-blame → Catastrophizing → Suicidal Behavior**	.038	.005
**Perceived Burdensomeness → Other Blame → Catastrophizing → Suicidal Behavior**	.054	.007
Effects from Thwarted belongingness to Suicidal Behavior		
**Total**	**.17**	.001
**Total Indirect**	.054	.12
**Thwarted Belongingness → Rumination → Suicidal Behavior**	-.012	.43
**Thwarted Belongingness → Catastrophizing → Suicidal Behavior**	.039	.072
**Thwarted Belongingness → Other Blame → Suicidal Behavior**	.007	.43
**Thwarted Belongingness → Self-blame → Rumination → Suicidal Behavior**	-.01	.81
**Thwarted Belongingness → Other Blame → Rumination → Suicidal Behavior**	.01	.16
**Thwarted Belongingness → Self-blame → Catastrophizing → Suicidal Behavior**	-.01	.81
**Thwarted Belongingness → Other Blame → Catastrophizing → Suicidal Behavior**	.02	.11
Effects from Self_Blame to Suicidal Behavior		
**Total**	.15	.001
**Total Indirect**	.15	.001
**Self-Blame → Rumination → Suicidal Behavior**	.052	.03
**Self-Blame → Catastrophizing → Suicidal Behavior**	.092	.001
Effects from Other Blam to Suicidal Behavior		
**Total**	.27	.001
**Total Indirect**	.21	.001
**Other blame → Rumination → Suicidal Behavior**	.054	.02
**Other Blame → Catastrophizing → Suicidal Behavior**	.15	.001

Bold Font: Indicates significant paths. Age, sex, and marital status were included as covariates. The coefficients are presented in the standardized format.

According to **Tables 3**, **4**, it is evident that thwarted belongingness exhibits a direct effect only on catastrophizing and suicidal behavior (p < .05). This suggests that individuals who perceive a lack of belongingness are more likely to experience catastrophizing and engage in suicidal behaviors.

Furthermore, the analysis indicates that the association between thwarted belongingness and suicidal behavior was not mediated through maladaptive components of cognitive-emotional regulation (p > .05). This implies that while thwarted belongingness directly influences catastrophizing and suicidal behavior, it does not operate through the pathways of maladaptive cognitive-emotional regulation strategies such as self-blame, rumination, catastrophizing, and other blame.

## Discussion

The interpersonal needs dimensions (i.e., perceived burdensomeness and thwarted belongingness) share the characteristic of being connected to emotional distress [e.g., 80], and individual differences in ER are transdiagnostic risk factors linked to psychopathology, which indicates individuals regulate their emotions and deal with emotional pain differently (81). From this angle, a study into associations between interpersonal needs and ER might aid in a clearer grasp of the reasons why people exhibit an upsurge in suicidal behavior (82).

The current study seeks to examine the direct and indirect pathways from perceived burdensomeness and thwarted belongingness to suicidal behavior through maladaptive ER strategies among an Iranian population. Our data and model modification appeared harmonious with previous studies demonstrating that interpersonal needs and maladaptive ER strategies are associated with suicidal behavior (12, 83). Those who strictly employ maladaptive strategies to regulate their emotions routinely go through prolonged and more intense episodes of distress or are more likely to engage in self-deteriorating behaviors [e.g., 84, 85]. In terms of direct effects, it was observed that both other blame and self-blame showed insignificance concerning suicidal behavior. This finding may seem somewhat unexpected. However, when examining the indirect effects, interesting pathways emerged through these strategies toward suicidal behavior.

The results suggest significant connections, revealing that perceived burdensomeness can lead to self-blame and other blame, which then contribute to rumination, ultimately culminating in suicidal behavior. This observation suggests that individuals who perceive themselves as a burden and attribute this perception to personal or external inadequacies may become trapped in a detrimental cycle of rumination, exacerbating their risk of suicidal behavior.

Previous findings in the literature indicate that self-blaming is a prevalent, dysfunctional practice that increases the risk of suicidal behavior (48), and it is proposed that perceived burdensomeness may be a manifestation of distress resulting in self-blame and other shame-related emotions (9).

Our research findings enrich the existing literature by providing a deeper comprehension of how maladaptive ER processes contribute to the pathways leading to suicidal behavior. While previous studies primarily focused on the presence of maladaptive strategies and cognitive content such as self-blame and other-blame, our research highlights the significance of repetitive negative thinking (i.e., rumination) as a crucial factor in driving individuals toward suicidal behavior. This perspective suggests a shift from merely acknowledging the existence of negative content to recognizing the detrimental impact of processing such content on psychopathological outcomes. This finding can also be understood within the framework of the cognitive attentive syndrome (86, 87), where negative content, such as feelings of burdensomeness, self-blame, or blaming others, when processed within a pattern of negative thinking (e.g., rumination), can result in adverse consequences. Indeed, according to the new waves of cognitive behavior therapy (e.g., acceptance and commitment therapy, dialectical behavior therapy), the way individuals process negative content can play a more detrimental role in psychopathology rather than the mere presence of negative content (88–90).

Internalized emotions of self-loathing, contempt, and burdensomeness may result in disengagement and isolation from others owing to a sense of not belonging (38). This is in line with previous research on the links between thwarted belongingness, perceived burdensomeness, and analogous constructs and suicidal behavior. For instance, Rogers et al. (2021) observed that thwarted belongingness accounted for the relationship between anger and suicidality, which is an emotion that is highly and maladaptively regulated by the other-blame strategy. Also, it has been found that perceived burdensomeness is responsible for the association between guilt and suicide risk, which is highly related to self-blame (38, 91).

Our findings highlight the complex interactions of perceived burdensomeness, self-blame, and other blame attributions, explaining how these variables interact to intensify catastrophic thinking and increase the risk of suicidal behavior. This finding is consistent with the larger framework of the cognitive attentive syndrome, as well as the tendency in the ruminative process that has been documented. In this context, the constant threat monitoring and the propensity to catastrophize internal/external incidents may amplify the already unsettling negative content (i.e., perceived burdensomeness, self-blame, other blame), making it harder to deal with and accelerating the emergence of psychopathological symptoms. These findings also could appropriately be explained by O’Connor et al.’s (2016) integrated motivational–volitional model of suicidal behavior (92). As per this theory, dysfunctional social conflicts and emotions of being knocked down (as a source of other blame) contribute to feelings of entrapment (a perceived incapacity to escape or be freed from uncomfortable situations)—and, eventually, suicidal thoughts and behavior.

In line with this Shim et al. (2017), examined the connection between perceived burdensomeness, catastrophizing, and suicide in relation to interpersonal needs. They showed that perceived burdensomeness mediates the link between pain catastrophizing and suicide (43).

In a seminal examination, Abdollahpour Ranjbar et al. (12) reported that depressed women with a history of suicide attempts use catastrophizing as an ER strategy more frequently than merely suicide ideators and healthy controls. However, catastrophizing as a cognitive distortion (i.e., excessive or unreasonable thinking pattern) and maladaptive ER strategy can be affected by other psychopathogenic thinking patterns. In the suicidal behavior context, it can include perceived burdensomeness and thwarted belongingness as cognitively distorted ways of perceiving individuals’ milieu and their interactions with others. Thus, additional research is required to investigate the mediating and moderating effects of catastrophizing in the context of perceived burdensomeness and thwarted belongingness, as well as how exaggerated and catastrophized these cognitions are seen by individuals at high risk of suicidal behavior.

Our finding for the mediating role of rumination between perceived burdensomeness and thwarted belongingness and suicidal behavior was found to be counterintuitive, and rumination did not mediate this association. Nevertheless, as mentioned above, our data provides a more nuanced mediating role for rumination. The results also indicated that rumination could mediate the association between self-blame and other blame with suicidal behavior. Roughly all of the previous studies account for a robust association between rumination and suicidal behavior (39). This finding aligns with the concept of “self-critical rumination” (93) or self-blame rumination, as described in the literature. Various studies have demonstrated its correlation with psychological difficulties, including depression, anxiety, and feelings of anger (94). Also, different studies showed the mediating role of rumination between other blame and adverse mental health outcomes like pathological personality traits (95) and suicidal ideation (96). Rogers et al. (2021) reported a strong relationship between suicide-specific rumination and suicidal intent, and this construct was connected to suicidal intent in addition to other suicide risk factors (e.g., thwarted belongingness, perceived burdensomeness, suicide ideation, etc.). As a result, greater research into this crucial overlooked field of study (i.e., probable associations between interpersonal needs and ruminative processes) in suicide studies appears to be more than essential.

### Clinical implications

Our results might have a number of clinical implications. Those who have high scores in perceived burdensomeness and thwarted belongingness and have a higher than regular inclination to blame others, ruminate, and catastrophize more can be more prone to suicidal behavior. Our findings may help clinicians think of these dispositions in their suicidal patients, with the potential therapeutic objective of enhancing supportive systems, amending cognitive distortions, and diminishing the use of maladaptive ER strategies that are found to be associated with disproportionate suicidal ideation and behavior. It expands the findings of Slee et al. (2008), who found that ER strategies are associated with suicidal behavior beyond and over depression (97). Accordingly, psychotherapeutic approaches that try to diminish suicidal behavior again may work better when they address both maladaptive ER strategies and depression symptoms.

Cognitive therapy, for instance, has shown efficacy in breaking the association between depressive symptoms and maladaptive cognitions, such as self-blame and worthlessness (98). Furthermore, psychotherapists may benefit from addressing specific suicide-related cognitions, such as low self-esteem and self-blame, to mitigate the risk of recurrent depressive episodes and chronic suicidal ideation (99).

In summary, while our study contributes to the growing body of literature on suicidal behavior, future research should aim to replicate and extend these findings in diverse populations and clinical settings. Adopting a cautious and nuanced approach to clinical interpretation will be essential in translating these findings into effective interventions for at-risk individuals.

### Limitations and further research

We were limited in how widely we could interpret our data because of the cross-sectional research design. In light of this, it is impossible to establish whether perceived burdensomeness and thwarted belongingness are causing suicidal behavior with the mediation of emotional processes. The use of just self-report assessments, which might be improved by more in-depth interviews and other measurements, is another potential drawback in our study. Additionally, cultural variations should be taken into account, at least for ER. Recent research has focused on the cultural differences in ER (100–102), and it is found that there are also differences in the Iranian culture [e.g., 103–106]. Future study endeavors can venture beyond cultural confines to investigate the practicality of the suggested framework. Examining gender variations within this paradigm may help us better understand how various groups’ manifestations of ER strategies and interpersonal needs vary.

In addition, concentrating on high-risk populations provides a chance to evaluate the prevalence and significance of interpersonal needs as well as how they interact with maladaptive emotion regulation in the intricate web of suicide conduct. Research projects of this kind may have the capacity to provide insights into focused treatments and preventative measures catered to the particular requirements of populations that are at risk. Finally, our data was imbalanced in terms of comprising a higher number of women. Thus, the generalizability of results should be of future research concern, which uses a proportionate number of genders and also more robust methodologies, including longitudinal designs.

## Conclusion

Our findings suggest that maladaptive cognitive ER strategies play a significant role in the contextual-interpersonal needs that underlie suicidal behavior. The therapeutic setting might use these findings to customize treatment interventions for those with suicidal behavior. As a result, we hypothesize that improving therapeutic outcomes would result from concentrating on the content of associated cognitions (i.e., perceived burdensomeness & thwarted belongingness), together with attempts to lessen the detrimental regulatory processes, such as rumination and catastrophization.

## Data availability statement

The raw data supporting the conclusions of this article will be made available by the authors, upon reasonable request.

## Ethics statement

The studies involving humans were approved by Maraghe University of Medical Sciences. The studies were conducted in accordance with the local legislation and institutional requirements. The participants provided their written informed consent to participate in this study.

## Author contributions

HAR: Study design, Conceptualization, Investigation, Project administration, Data curation, Methodology, Formal analysis, Validation, Visualization, Writing – original draft, Writing – review & editing, Resources. MB-B: Data curation, Investigation, Project administration, Methodology, Resources, Writing – review & editing. SF-N: Data curation, Project administration, Resources, Writing – review & editing. IH: Methodology, Validation, Visualization, Writing – review & editing. MHA: Supervision, Conceptualization, Methodology, Data curation, Visualization, Formal analysis, Writing – original draft, Writing – review & editing. ME: Supervision, Conceptualization, Writing – review & editing, Validation, Visualization.
